# Intervention Effect of Oropharyngeal Administration of Colostrum in Preterm Infants: A Meta-Analysis

**DOI:** 10.3389/fped.2022.895375

**Published:** 2022-06-27

**Authors:** Mengyue Huo, Chunli Liu, Hua Mei, Yuheng Zhang, Chunzhi Liu, Dan Song, Yayu Zhang, Yanbo Zhang, Chun Xin

**Affiliations:** Department of Neonatology, Affiliated Hospital, Inner Mongolia Medical University, Hohhot, China

**Keywords:** preterm infants, meta-analysis, oropharyngeal administration, colostrum, infant

## Abstract

**Objective:**

To evaluate the efficacy and safety of oropharyngeal administration of colostrum (OAC) in preterm infants.

**Methods:**

We searched Embase, MEDLINE, Cochrane Central Register of Controlled Trials (CENTRAL), Cumulative Index to Nursing and Allied Health Literature (CINAHL), and the website of the clinical trials, search time was from the establishment of the databases or websites up to 1 February 2022. Preterm infants with gestational age (GA) ≤ 32 weeks or birth weight (BW) ≤ 1500 g were taken as the participants, collect randomized controlled trials (RCTs) of comparing OAC and placebo or no intervention in preterm infants. Two researchers independently screened the literature, extracted the data, and evaluated the quality of the literature, and we adopted Review Manager 5.3 software for meta-analysis.

**Results:**

In total, 11 RCTs (*n* = 1,173) were included in the review. A meta-analysis showed significant difference in the incidence of necrotizing enterocolitis [NEC; *p* = 0.009, relative ratio (RR) = 0.51, 95% confidence interval (CI) = 0.31–0.84], late-onset sepsis (LOS; *p* = 0.02, RR = 0.75, 95% CI = 0.59–0.95), ventilator-associated pneumonia (VAP; *p* = 0.03, RR = 0.48, 95% CI = 0.24–0.95), the time to reach full enteral feeds (*p* < 0.00001, mean difference (MD) = −3.40, 95% CI = −3.87 to −2.92), duration of hospital stay (*p* < 0.00001, MD = −10.00, 95% CI = −11.36 to −8.64), and the rate of weight gain (kg.d; *p* < 0.00001, MD = 2.63, 95% CI = 2.10–3.16) between the colostrum group and control group. Meanwhile, researchers found no significant difference between the colostrum group and control group in the incidence of bronchopulmonary dysplasia (BPD; *p* = 0.17, RR = 0.83, 95% CI = 0.64–1.08), intraventricular hemorrhage (IVH; grade ≥3; *p* = 0.05, RR = 0.44, 95% CI = 0.19–1.01), periventricular leukomalacia (PVL; *p* = 0.67, RR = 0.70, 95% CI = 0.14–3.49), retinopathy of prematurity (ROP; *p* = 0.29, RR = 1.25, 95% CI = 0.82–1.89), and patent ductus arteriosus (PDA; *p* = 0.17, RR = 1.22, 95% CI = 0.92–1.62).

**Conclusion:**

Oropharyngeal administration of colostrum can reduce the incidence of NEC, LOS, and VAP in preterm infants, shortening the time to reach full enteral feeds, and duration of hospital stay, and increasing the rate of weight gain (kg.d). Therefore, OAC can be used as part of routine care for preterm infants.

## Introduction

As we all know, colostrum is the best natural food for a baby. Colostrum is not only rich in nutrition, with an appropriate proportion of sugar, fat, and protein, which is conducive to digestion and absorption, but also rich in immune factors, such as secretory immunoglobulin A (SIgA), lactoferrin, bifidus factor, and lysozyme, which can regulate immunity, anti-infection, and sterilization (1). With the rapid development of perinatal medicine, the survival rate of preterm infants is increasing year by year, especially very preterm infants and very low birth weight (BW) infants. During the period of hospitalization in the neonatal intensive care unit (NICU), the management of the nutritional enteral diet of preterm infants is often limited due to an immature digestive system, delayed intestinal feeding, or the use of antibiotics, resulting in flora imbalance, which greatly increases necrotizing enterocolitis (NEC), late-onset sepsis (LOS), neonatal mortality, and so on (2). Therefore, neonatologists urgently need to find a safe and effective alternative to inject preterm colostrum into preterm infants when they cannot be fed enterally in the early postnatal period. Rodriguez et al. proposed oropharyngeal administration of colostrum (OAC), that is to say, a small amount of colostrum is evenly applied to the oral mucosa on both sides with a syringe, cotton swab, or oral applicator, and the immune effect is achieved through the absorption of the oral mucosa, thereby reducing the risk of infection and helping the colonization of oral beneficial bacteria (3). It is a colostrum supplement for preterm infants who cannot eat orally or fast. Aggarwal et al. randomly divided 260 newborns with the gestational age of 26–31 weeks into two groups; they received 0.2 ml of human milk or placebo respectively *via* the oropharyngeal route, beginning within 24 h after birth, and continued every 3 h until oral feeds were initiated. It was found that a strategy of OAC in very preterm and extremely preterm infants did not decrease the composite primary outcome of death, LOS, or NEC (4). However, a randomized controlled study in China shows that for the preterm infants with gestation age (GA) ≤ 32 weeks, OAC yields a potential effect in decreasing the incidences of NEC, LOS, and severe intraventricular hemorrhage (IVH) (5). In view of the great differences between the results of various studies, a relatively unified opinion has not yet been formed, this study intends to systematically retrieve and evaluate the impact of OAC on preterm infants, which is expected to be helpful to neonatologists in clinical practice.

## Data and Methods

### Literature Inclusion Criteria

(1) Types of participants: Preterm infants with BW ≤1,500 g or gestational age ≤32 weeks were included in the review.
(2) Types of studies: We included all published clinical randomized controlled trials (RCTs), and articles are written in English.
(3) Types of intervention: The subjects in the colostrum group were given OAC, and the subjects in the control group were given placebo or routine care.
(4) Types of outcome measures: Describe at least one primary or secondary outcome index set in this systematic analysis.

### Literature Exclusion Criteria

(1) Non-human studies, reviews, commentaries, editorials, abstracts, letters, and so on.
(2) Included subjects were literature on severe congenital malformations.
(3) Republished literature.
(4) Literature with unclear data description, wrong statistical methods, or data provided that cannot be analyzed by meta-analysis.

### Outcome of Review

#### Primary Outcomes

(1) Incidence of NEC.
(2) Incidence of LOS.
(3) Death before discharge to home.

#### Secondary Outcomes

(1) Time to reach full enteral feeds.
(2) Duration of hospital stay.
(3) Incidence of bronchopulmonary dysplasia (BPD).
(4) Incidence of ventilator-associated pneumonia (VAP).
(5) Incidence of IVH (grade ≥3).
(6) Incidence of periventricular leukomalacia (PVL).
(7) Incidence of retinopathy of prematurity (ROP).
(8) Incidence of patent ductus arteriosus (PDA).
(9) Rate of weight gain (kg.d).

### Literature Search Strategy

We conducted a comprehensive search that included the Embase, MEDLINE, Cochrane Central Register of Controlled Trials (CENTRAL), Cumulative Index to Nursing and Allied Health Literature (CINAHL), and the website of the clinical trials, search time from the establishment of the databases or websites was up to 1 February 2022. We searched using Medical Subject Headings (MeSH) terms: “preterm infant”[MeSH] OR “premature infant” [MeSH] OR “Infant, newborn” [MeSH] OR “Infant” [MeSH] OR “infant, low birthweight” [MeSH] OR “Infant, Very Low Birth Weight” [MeSH] OR “Infant, Extremely Low Birth Weight” [MeSH] OR “Infant, Extremely Premature” [MeSH] OR “Very premature infants” [MeSH] AND “Colostrum” [MeSH] OR “oropharyngeal administration.”

### Study Selection

We followed the standard processes recommended by the Cochrane Handbook for Systematic Reviews of Interventions. Two of the review investigators independently screened the titles and abstracts of all of the relevant studies identified by the search. After that, full-text articles were downloaded, read, and assessed using the confirmed criteria for exclusion or inclusion. Any disagreement between the researchers on the inclusion or exclusion of a paper was settled by consensus or by inviting a third reviewer.

### Data Extraction

Two review authors independently extracted and compared data, in case of disagreement, the two reviewers settled it through consensus or by inviting a third reviewer. Last, we extracted the following data from each study.

1. Literature basic characteristics (author, year, country, random sequence generation, allocation concealment, and blinding).
2. Participants (sample size, GA, and BW).
3. Intervention (technique, dosage, interval, duration, and any additional interventions).
4. Primary outcomes (incidence of NEC, incidence of LOS, and death before discharge to home).
5. Secondary outcomes (time to reach full enteral feeds, duration of hospital stay, the incidence of BPD, VAP, IVH (grade ≥3), PVL, ROP, PDA, and rate of weight gain (kg.d).

### Risk of Bias Assessment

Two review authors according to the methods in the Cochrane Handbook for Systematic Reviews of Interventions independently assessed the risk of bias (low, high, or unclear) of all included trials. Total of six domains were evaluated and categorized into low risk, high risk, and unclear risk. These six domains were random sequence generation, allocation concealment, blinding of participants and personnel, blinding of outcome data, incomplete outcome data, and selective reporting.

### Statistical Analysis

A meta-analysis was performed using the Review Manager (version 5.3). Firstly, the heterogeneity of the literature was tested. When *I*^2^ ≤ 50%, the fix-effect model was used for analysis, in which *I*^2^ < 25% means that there is no heterogeneity between the collected documents, and when 25% ≤ *I*^2^ ≤ 49%, it means that the heterogeneity between the collected documents is low. When *I*^2^ > 50%, it indicates that there is heterogeneity in the collected literature, of which 50% ≤ *I*^2^ ≤ 74%, it means that there is moderate heterogeneity between the collected documents, and when *I*^2^ > 75%, it means that the heterogeneity between the collected documents is high, the source of heterogeneity was further analyzed. After excluding the influence of obvious clinical heterogeneity, the random effects model was used for analysis. The relative ratio (RR) was used as the effect index for the counting data, and the standardized difference (SMD) was used as the effect index for the measurement data. For each effect quantity, given its point estimate and 95% confidence interval (CI). *p* < 0.05 was statistically significant.

## Results

### Study Selection and Characteristics

**Figure 1:**
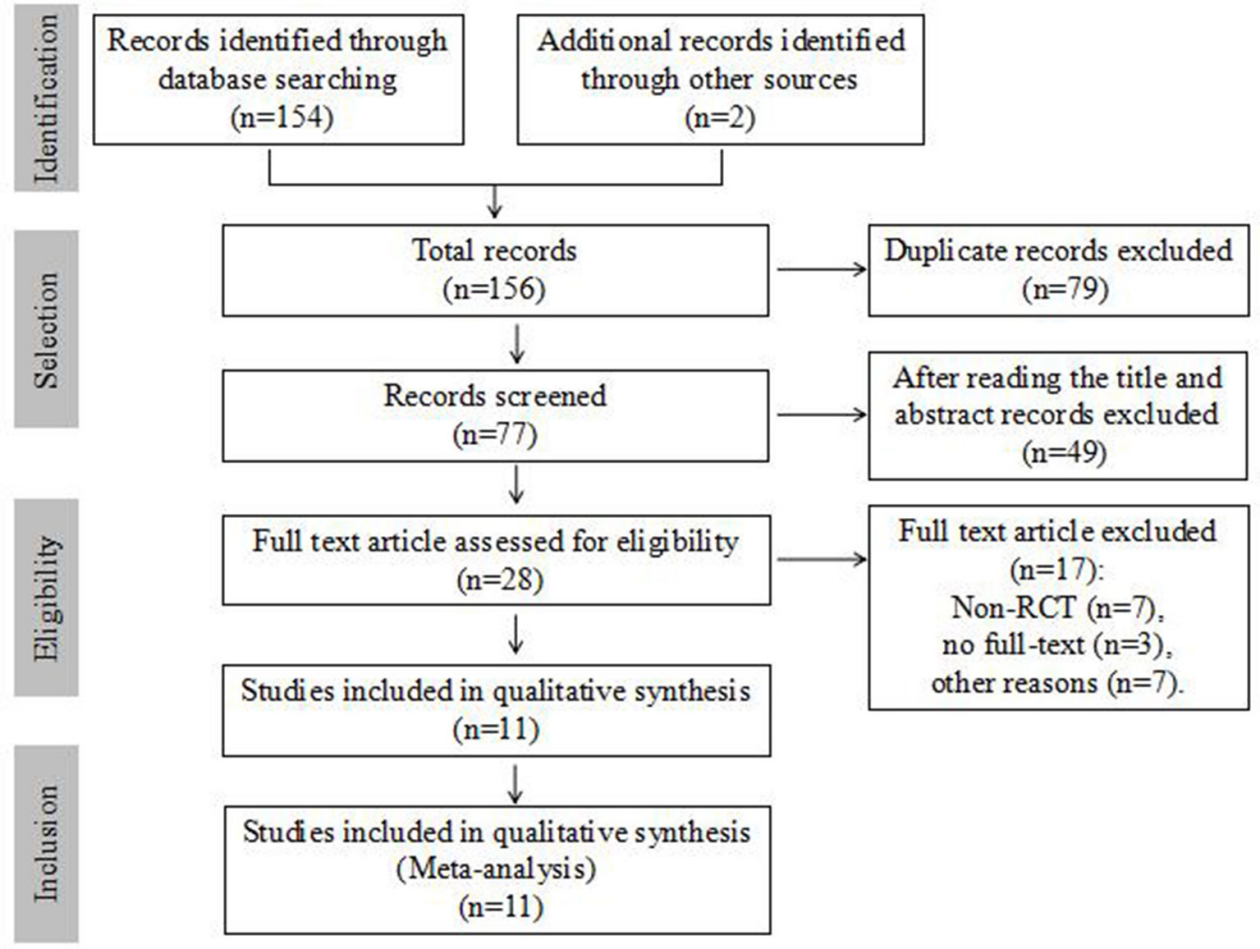
Flow diagram of study selection.

Initially, 156 records were identified through database searching and other sources, 79 duplicate studies were excluded, and 49 were excluded after reading the title and abstract. Then, 17 were excluded after full-text reading, i.e., 7 non-RCT studies, 3 unable to obtain the full text, and 7 for other reasons. Finally, 11 RCTs met the inclusion criteria of this review. Among them, 581 in colostrum group and 592 in control group, a total of 1,173 preterm infants were included (4–14). The study selection process is shown in Figure 1. The details of the included studies are summarized in Table 1.

**Table 1:** General characteristics of included studies.

**Author, country, Methods**	**Sample size (colostrum /controls)**	**GA**		**BW**		**Interventions**		**Outcome indicators**
		**Colostrum group**	**Control group**	**Colostrum group**	**Control group**	**Colostrum group**	**Control group**	
Aggarwal et al. (4), India, RCT	117/115	30 (28–31)^b^	30 (29–31)^b^	1,205 ± 297^a^	1,198 ± 259^a^	0.2 ml of maternal colostrum was administered through oropharynx every 3 h, until oral feeds were initiated	0.2 mL of sterile water was applied as in colostrum group	a, b, c, d, e, f, g, h, j, k,
OuYang et al. (5), China, RCT	127/125	30.00 ± 1.83^a^	29.65 ± 2.04^a^	1,302.26 ± 209.81^a^	1,328.72 ± 222.17^a^	0.4 ml of maternal colostrum was administered through oropharynx every 3 h, lasted for a total of 10 days.	0.4 mL of normal saline was applied as in colostrum group	a, b, c, d, e, f, h, i, j, k, l
Abd-Elgawad et al. (6), Egypt, RCT	100/100	28.9 ± 2.05^a^	28.8 ± 2.26^a^	1,050 ± 246^a^	1,022 ± 249^a^	0.2 ml of mother's colostrum was administered through oropharynx every 2–4 h, until reached complete enteral feeding.	Nothing was administered during the pre-feeding period.	a, b, c, d, e, f, g, l
Ferreira et al. (7), Brazil, RCT	47/66	28 (27–30)^b^	28 (26–31)^b^	1,048 (787–1,217)^b^	1,036 (801–1,206)^b^	0.2 ml of maternal colostrum was administered through oropharynx every 2 h, lasted for a total of 2 days.	0.2 mL of sterile water was applied as in colostrum group	a, b, c, f, h, j,
Sharma et al. (8), India, RCT	59/58	29.1 ± 1.8^a^	29.2 ± 1.9^a^	1,146 ± 58^a^	1,158 ± 61^a^	0.2 ml of maternal colostrum was administered through oropharynx every 2 h, lasted for a total of 3 days.	Routine care.	a, b, c, d, e, f, g, i, j,
Romano-Keeler et al. (9), America, RCT	48/51	30 (27, 31)^b^	29 (28, 30)^b^	1,272 (988, 1,602)^b^	1,170 (905, 1,340)^b^	0.2 ml of maternal colostrum was administered through oropharynx every 6 h, lasted for a total of 5 days.	Routine care.	a, b, c,
Glass et al. (10), America, RCT	17/13	28.4 ± 0.7^a^	28.5 ± 0.8^a^	1,132 ± 64^a^	1,079 ± 59^a^	0.2 ml of maternal colostrum was administered through oropharynx every 3 h, from 2 to 7 days.	0.2 mL of sterile water was applied as in colostrum group	a, b, c, d,
Zhang et al. (11), China, RCT	27/28	29.86 ± 2.02^a^	30.46 ± 2.50^a^	1,241 ± 275^a^	1,248 ± 233^a^	0.2 ml of colostrum was administered through oropharynx every 4 h, lasted for a total of 3 days.	0.2 mL of sterile water was applied as in colostrum group	a, b, c, d,
Sohn et al. (12), America, RCT	6/6	27 (25–30)^b^	27 (25–28)^b^	1,092 (490–1,350)^b^	1,015 (735–1,300)^b^	0.2 ml of colostrum was administered through oropharynx every 2 h, lasted for a total of 46 h.	Routine care.	a, b, c, f, g,
Lee et al. (13), Korea, RCT	24/24	26 ^+5^ (24^+2^-27^+4^)^b^	26^+5^ (24^+3^-27^+1^)^b^	830 (701–993)^b^	815 (610–1,003)^b^	0.2 ml of maternal colostrum was administered through oropharynx every 3 h, lasted for a total of 3 days.	0.2 mL of sterile water was applied as in colostrum group.	a, b, c, f, g, h, j,
Nancy and Rodriguez (14), America, RCT	9/6	25.97 ± 1.00^a^	26.77 ± 0.97^a^	776.11 ± 231.73^a^	940.83 ± 181.34^a^	0.2 ml of maternal colostrum was administered through oropharynx every 2 h, lasted for a total of 2 days.	0.2 mL of sterile water was applied as in colostrum group	a, b, c, d, e,

*BW, birth weight; GA, gestation age; (a) incidence of NEC, (b) incidence of LOS, (c) death before discharge to home, (d) time to reach full enteral feeds, (e) duration of hospital stay, (f) incidence of BPD, (g) incidence of VAP, (h) incidence of (IVH) (grade ≥3), (i) incidence of PVL, (j) incidence of ROP, (k) incidence of PDA, (l) rate of weight gain (kg.d),*

a *mean ± standard deviation (SD),*

b *median (interquartile range)*.

### Risk of Bias in Included Studies

**Figure 2:**
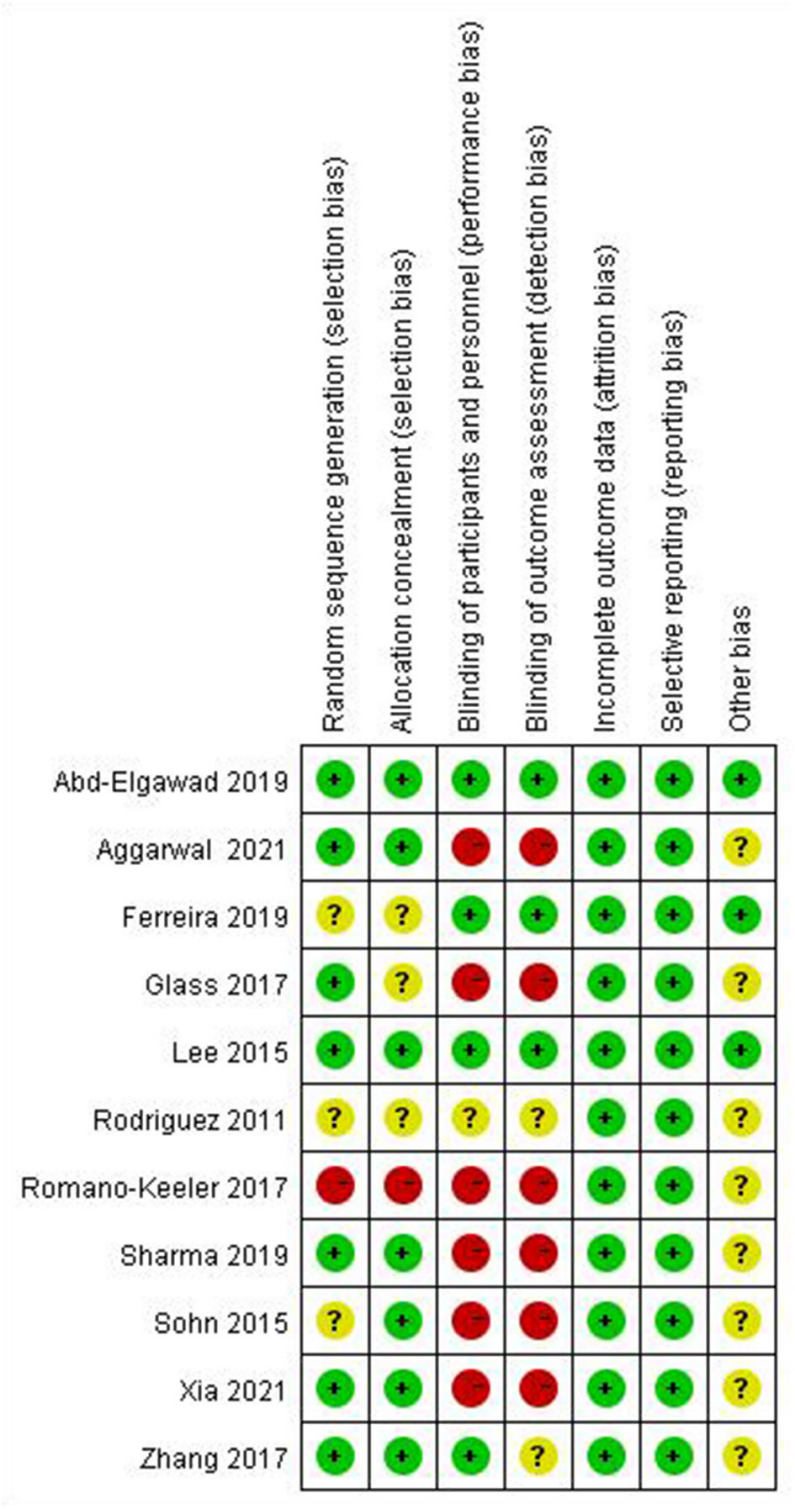
Summary of risk of bias.

**Figure 3:**
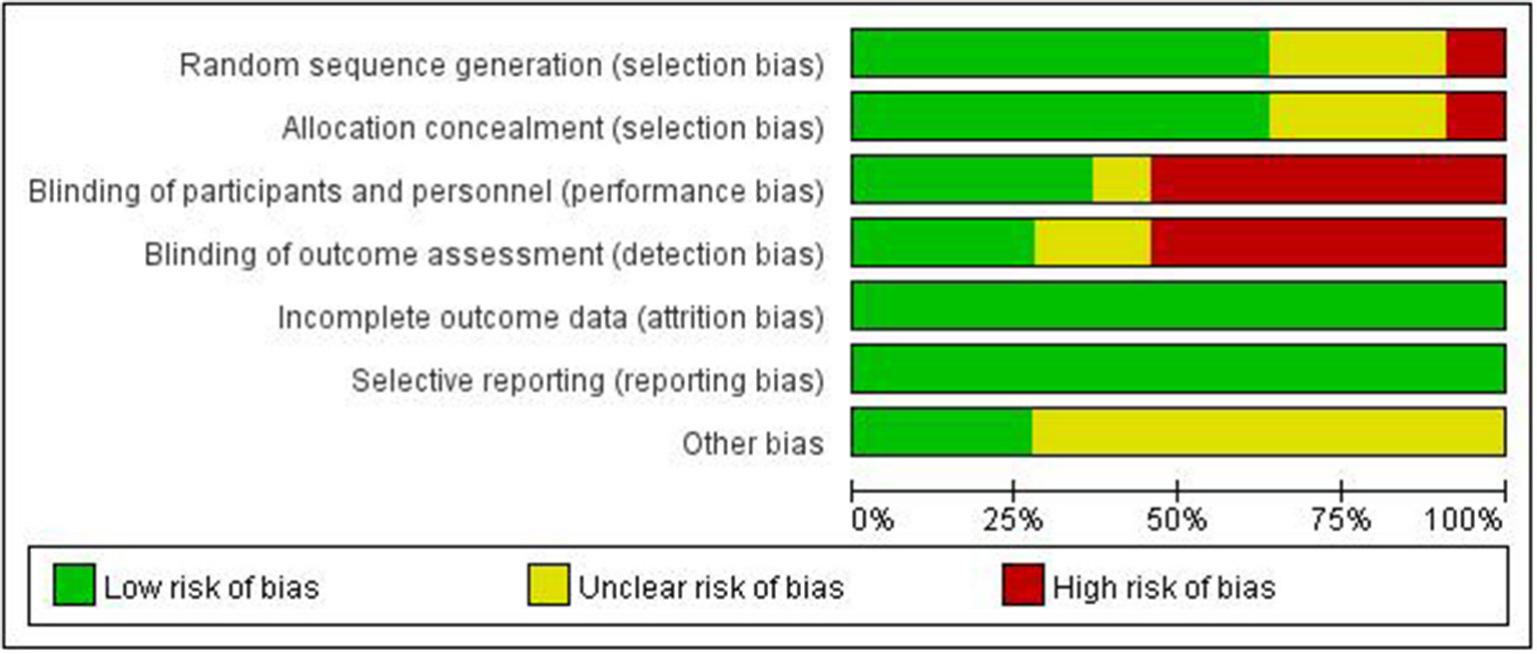
Summary of risk of bias.

We adopted the methods in the Cochrane Handbook for Systematic Reviews of Interventions to assess the risk of bias (low, high, or unclear) of all included studies. The results of the risk of bias assessment are shown in Table 2, Figures 2, 3.

**Table 2:** Risk of bias in included studies.

**Studies**	**Random sequence generation**	**Allocation concealment**	**Blinding of participants and personnel**	**Blinding of outcome assessment**	**Incomplete outcome data**	**Selective reporting**	**Other bias**
Aggarwal et al. (4)	Low risk	Low risk	High risk	High risk	Low risk	Low risk	Unclear risk
OuYang et al. (5)	Low risk	Low risk	High risk	High risk	Low risk	Low risk	Unclear risk
Abd-Elgawad et al. (6)	Low risk	Low risk	Low risk	Low risk	Low risk	Low risk	Low risk
Ferreira et al. (7)	Unclear risk	Unclear risk	Low risk	Low risk	Low risk	Low risk	Low risk
Sharma et al. (8)	Low risk	Low risk	High risk	High risk	Low risk	Low risk	Unclear risk
Romano-Keeler et al. (9)	High risk	High risk	High risk	High risk	Low risk	Low risk	Unclear risk
Glass et al. (10)	Low risk	Unclear risk	High risk	High risk	Low risk	Low risk	Unclear risk
Zhang et al. (11)	Low risk	Low risk	Low risk	Unclear risk	Low risk	Low risk	Unclear risk
Sohn et al. (12)	Unclear risk	Low risk	High risk	High risk	Low risk	Low risk	Unclear risk
Lee et al. (13)	Low risk	Low risk	Low risk	Low risk	Low risk	Low risk	Low risk
Nancy and Rodriguez (14)	Unclear risk	Unclear risk	Unclear risk	Unclear risk	Low risk	Low risk	Unclear risk

### Primary Outcomes

#### The Incidence of NEC

**Figure 4:**
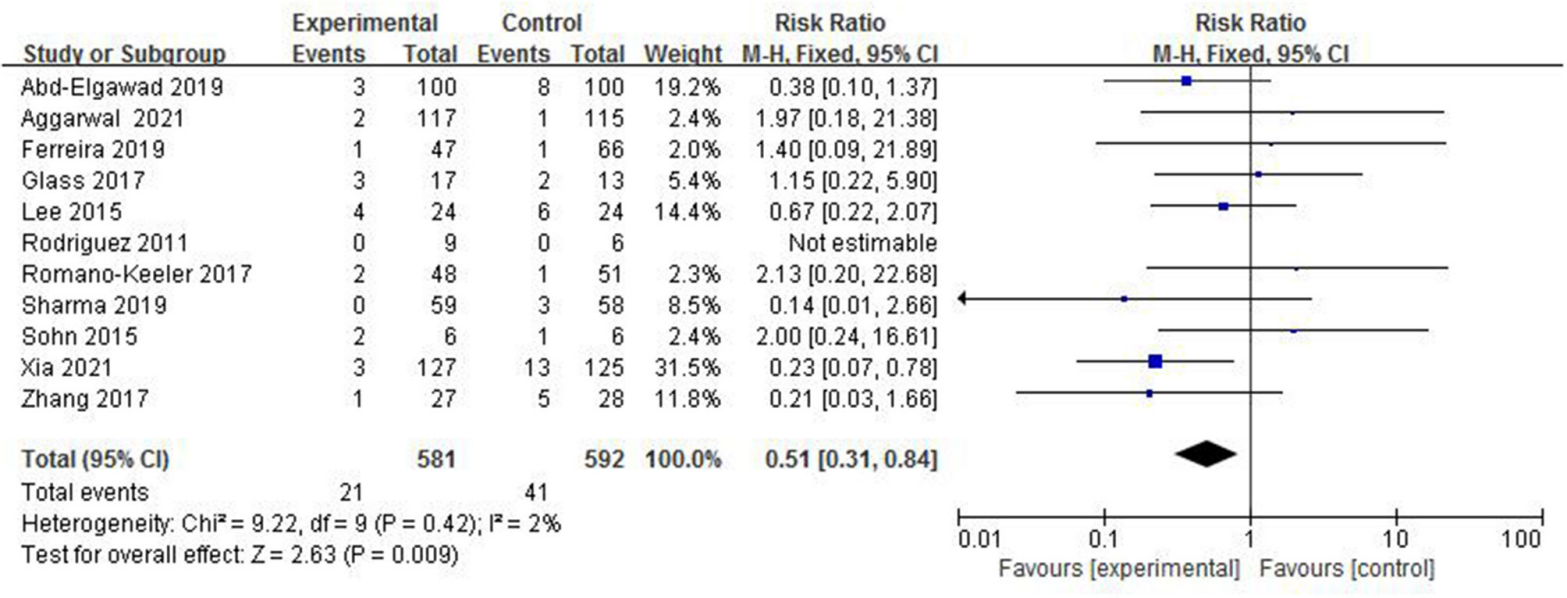
Forest plot of the incidence of NEC.

Necrotizing enterocolitis is a common serious gastrointestinal disease in newborns. The main clinical manifestations are abdominal distension, vomiting, and bloody stool. All included studies (4–14) reported the incidence of NEC in 1,173 preterm infants (Figure 4). The incidence of NEC in the colostrum group was 3.6% (21/581) and in the control group was 6.9% (41/592). The heterogeneity test (*I*^2^ = 2%, *p* = 0.42) suggested that there was homogeneity among the studies, therefore, the fix-effect model was used for statistical analysis. A meta-analysis showed that there was significant difference in the incidence of NEC between the colostrum group and control group (*p* = 0.009, RR = 0.51, 95% CI = 0.31–0.84).

#### The Incidence of LOS

**Figure 5:**
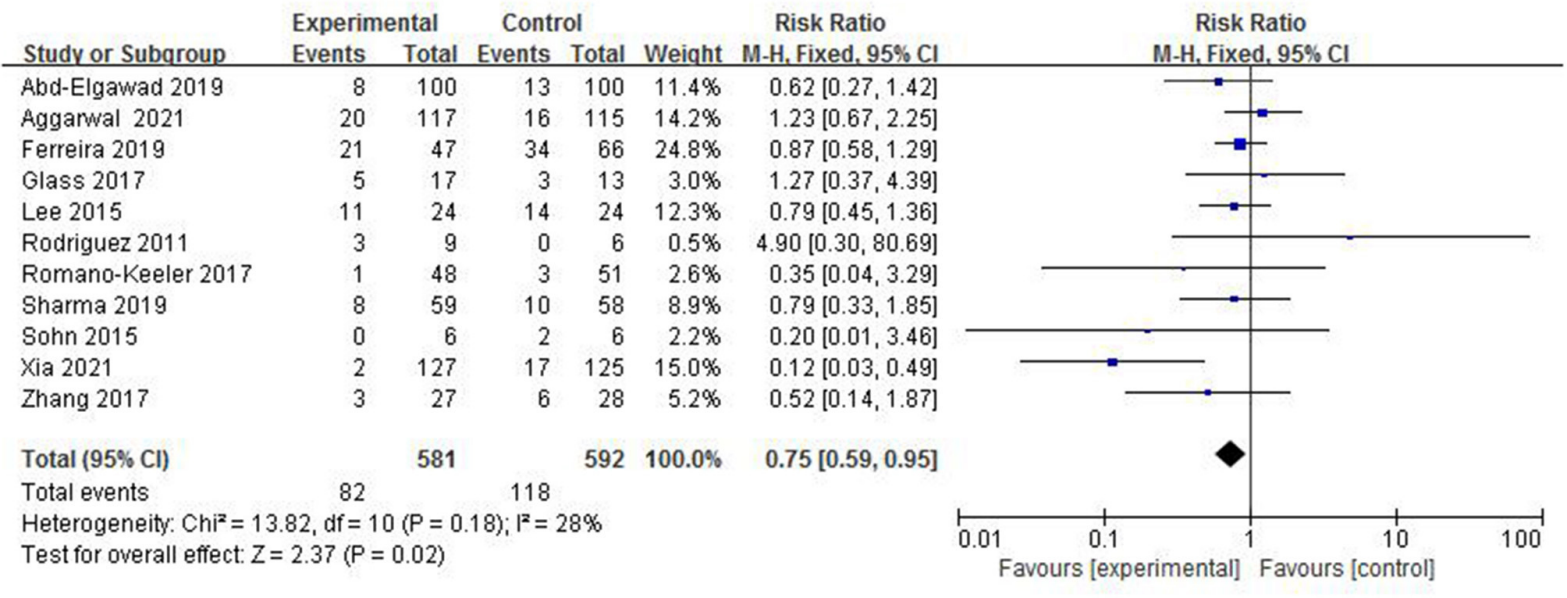
Forest plot of the incidence of LOS.

Late-onset sepsis is a serious infectious disease in the neonatal period. The systemic inflammatory reaction is caused by pathogens that invade the blood of newborns and grow, reproduce, and produce toxins. In total, 11 studies (4–14), with a total sample of 1,173 preterm infants, measured the incidence of LOS (Figure 5). The incidence of LOS in the colostrum group was 14.1% (82/581) and in the control group was 19.9% (118/592). The fixed-effect model was used due to a low heterogeneity among studies (*I*^2^ = 28%, *p* = 0.18). A meta-analysis showed that there was significant difference in the incidence of LOS between the colostrum group and control group (*p* = 0.02, RR = 0.75, 95% CI = 0.59–0.95).

#### Death Before Discharge to Home

**Figure 6:**
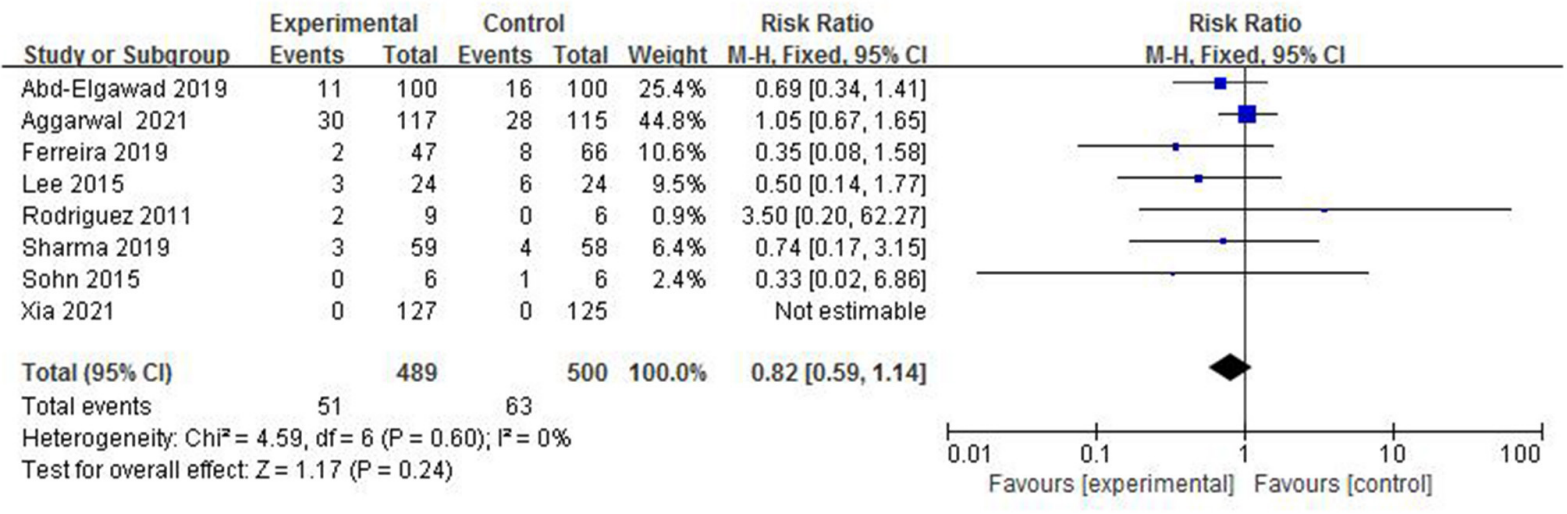
Forest plot of the death before discharge to home.

In total, 8 studies (4–8, 12–14) reported death before discharge to home in 989 preterm infants (Figure 6). The rate of death before discharge to home in the colostrum group was 10.4% (51/489) and in the control group was 12.6% (63/500). The fix-effect model was used for statistical analysis because the heterogeneity test (*I*^2^ = 0%, *p* = 0.60) suggested that there was homogeneity among the studies. A meta-analysis showed there was no significant difference in the rate of death before discharge to home between the colostrum group and control group (*p* = 0.24, RR = 0.82, 95% CI = 0.59–1.14).

### Secondary Outcomes

#### Time to Reach Full Enteral Feeds

**Figure 7:**
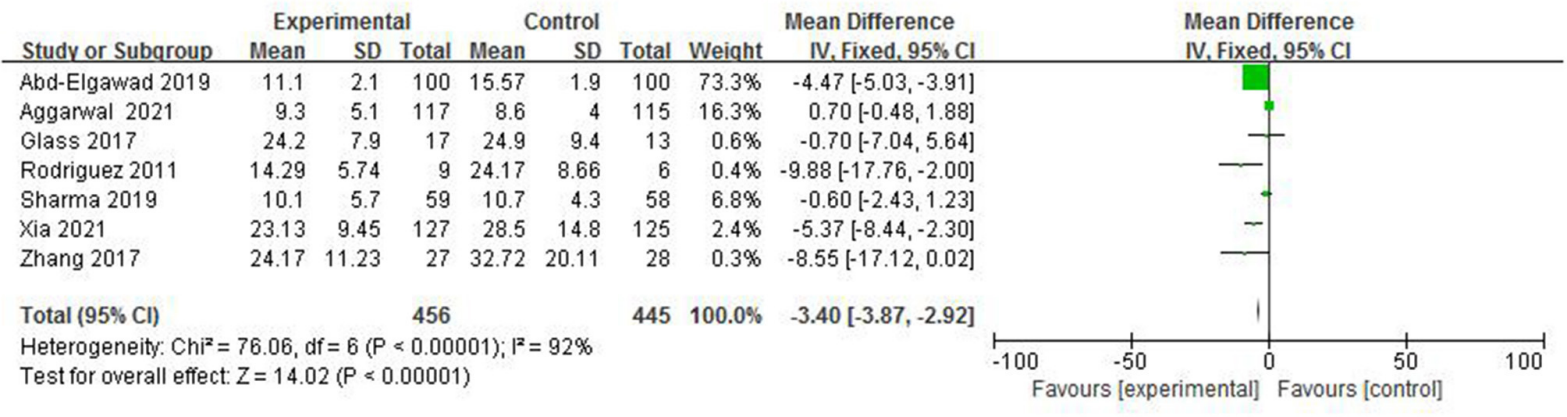
Forest plot of time to reach full enteral feeds.

In total, 7 studies (4–6, 8, 10, 11, 14) reported the time to reach full enteral feeds in 901 preterm infants (Figure 7). The random-effect model was used to estimate the combined result due to the strong heterogeneity among studies (*I*^2^ = 92%, *p* < 0.00001). A meta-analysis showed there was significant difference in the time to reach full enteral feeds between the colostrum group and control group (*p* < 0.00001, MD = −3.40, 95% CI = −3.87 to −2.92).

#### Duration of Hospital Stay

**Figure 8:**
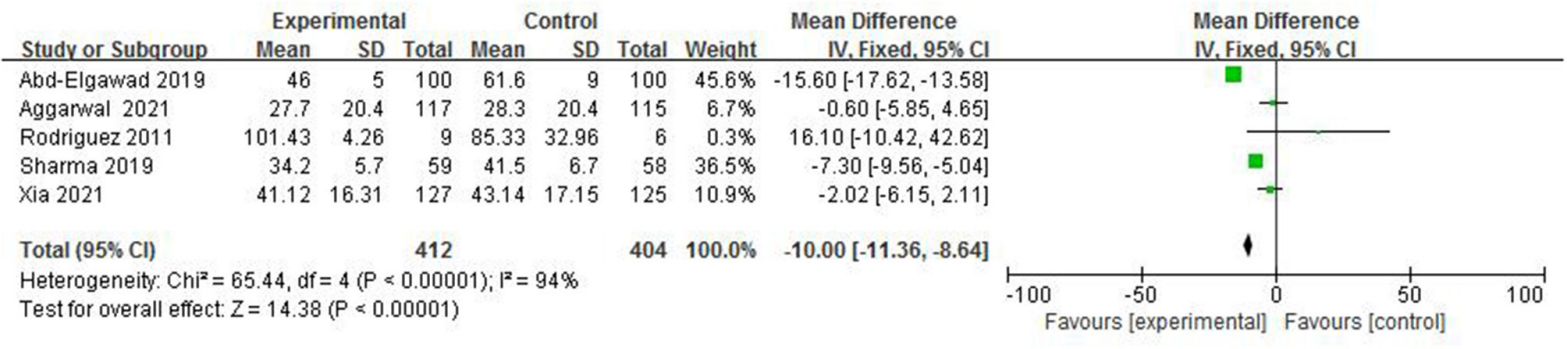
Forest plot of duration of hospital.

In total, five studies (4–6, 8, 14) reported the duration of hospital stay in 816 preterm infants (Figure 8). The random-effect model was used to estimate the combined result because of the strong heterogeneity among studies (*I*^2^ = 94%, *p* < 0.00001). A meta-analysis showed that there was significant difference in duration of hospital stay between the colostrum group and control group (*p* < 0.00001, MD = −10.00, 95% CI = −11.36 to −8.64).

#### The Incidence of BPD

**Figure 9:**
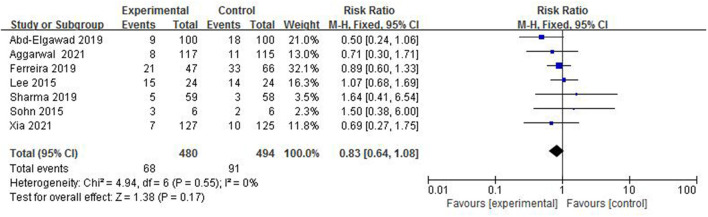
Forest plot of the incidence of BPD.

Bronchopulmonary dysplasia is a common complication of premature infants. The current definition is that premature infants still need oxygen at 28 days after birth or 36 weeks after correcting gestational age. In total, 7 studies (4–8, 12, 13) reported the incidence of BPD in 974 preterm infants (Figure 9). The incidence of BPD in the colostrum group was 14.2% (68/480) and in the control group was 18.4% (91/494). The heterogeneity test (*I*^2^ = 0%, *p* = 0.55) suggested that there was homogeneity among the studies, therefore, the fix-effect model was used for statistical analysis. A meta-analysis showed that there was no significant difference in the incidence of BPD between the colostrum group and control group (*p* = 0.17, RR = 0.83, 95% CI = 0.64–1.08).

#### The Incidence of VAP

**Figure 10:**
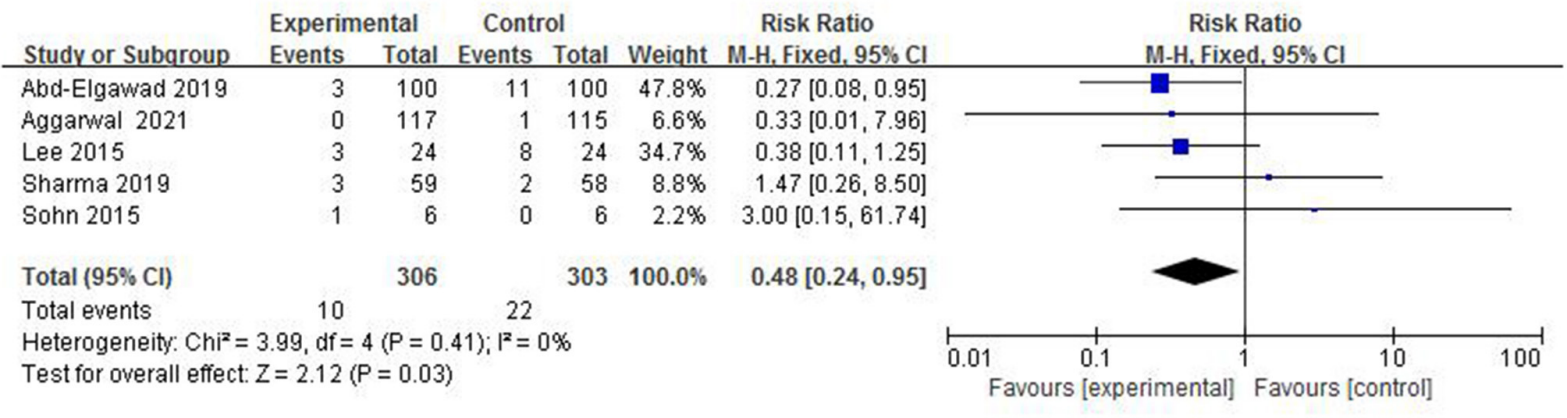
Forest plot of the incidence of VAP.

Ventilator-associated pneumonia is the main acquired infection in NICU, which is pulmonary inflammation 48 h after mechanical ventilation. In total, 5 studies (4, 6, 8, 12, 13) reported the incidence of VAP in 609 preterm infants (Figure 10). The incidence of VAP in the colostrum group was 3.3% (10/306) and in the control group was 7.3% (22/303). The fix-effect model was used for statistical analysis because of the heterogeneity test (*I*^2^ = 0%, *p* = 0.41) suggesting that there was homogeneity among the studies. A meta-analysis showed that there was significant difference in the incidence of VAP between the colostrum group and control group (*p* = 0.03, RR = 0.48, 95% CI = 0.24–0.95).

#### The Incidence of IVH (Grade ≥3)

**Figure 11:**
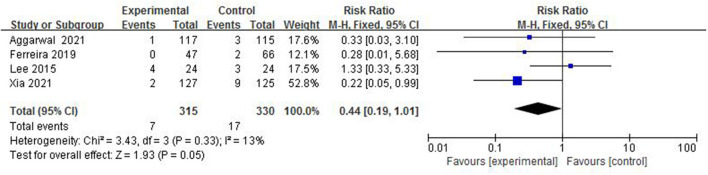
Forest plot of the incidence of IVH (grade ≥3).

Intraventricular hemorrhage is a common type of intracranial hemorrhage in preterm infants, and it is also one of the main causes of death and disability in preterm infants. In total, 4 studies (4, 5, 7, 13) reported the incidence of IVH (grade ≥3) in 645 preterm infants (Figure 11). The incidence of IVH in the colostrum group (grade ≥3) was 2.2% (7/315) and in the control group was 5.2% (17/330). The fix-effect model was used for statistical analysis because of the heterogeneity test (*I*^2^ = 13%, *p* = 0.33) suggesting that there was homogeneity among the studies. A meta-analysis showed that there was no significant difference in the incidence of IVH (grade ≥3) between the colostrum group and control group (*p* = 0.05, RR = 0.44, 95% CI = 0.19–1.01).

#### The Incidence of PVL

**Figure 12:**
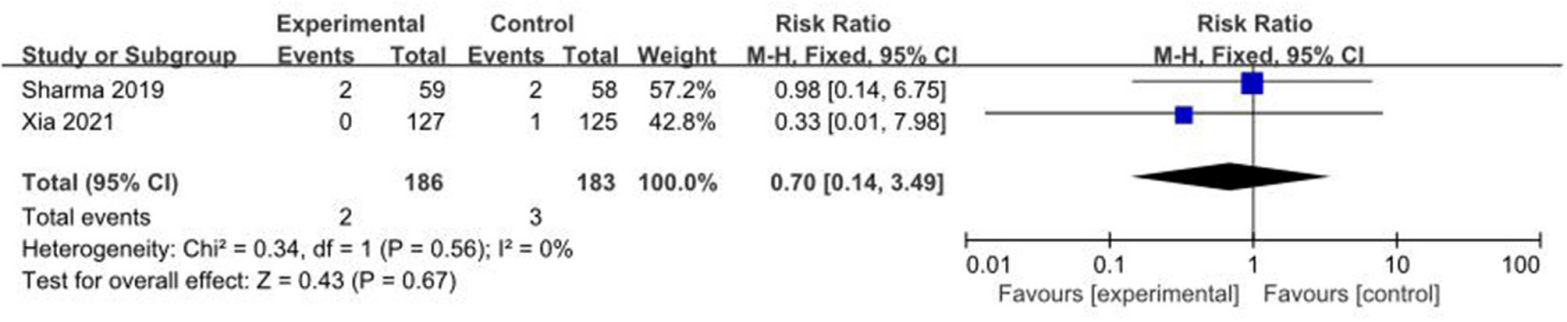
Forest plot of the incidence of PVL.

White matter injury is one of the unique forms of brain injury in premature infants. The most serious outcome is PVL, which causes neurological sequelae in children. In total, 2 studies (5, 8), with a total sample of 369 preterm infants, measured the incidence of PVL (Figure 12). The incidence of PVL in the colostrum group was 1.1% (2/186) and in the control group was 1.6% (3/183). The fixed-effect model was used due to a low heterogeneity among studies (*I*^2^ = 0%, *p* = 0.56). A meta-analysis showed that there was no significant difference in the incidence of PVL between the colostrum group and control group (*p* = 0.67, RR = 0.70, 95% CI = 0.14–3.49).

#### The Incidence of ROP

**Figure 13:**
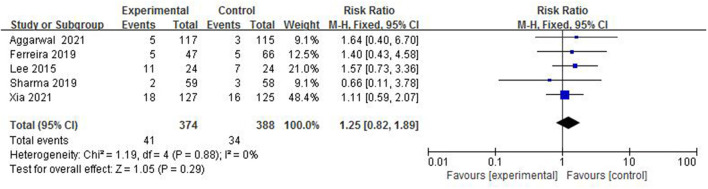
Forest plot of the incidence of ROP.

Retinopathy of prematurity is an important disease that leads to blindness or severe visual impairment in preterm infants. In total, 5 studies (4, 5, 7, 8, 13), with a total sample of 762 preterm infants, measured the incidence of ROP (Figure 13). The incidence of ROP in the colostrum group was 11.0% (41/374) and in the control group was 5.8% (34/588). The fixed-effect model was used due to a low heterogeneity among studies (*I*^2^ = 0%, *p* = 0.88). A meta-analysis showed that there was no significant difference in the incidence of ROP between the colostrum group and the control group (*p* = 0.29, RR = 1.25, 95% CI = 0.82–1.89).

#### The Incidence of PDA

Patent ductus arteriosus is one of the common congenital heart diseases and one of the main causes of heart failure in premature infants. In total, 2 studies (4, 5) reported the incidence of PDA in 484 preterm infants. The incidence of PDA in the colostrum group was 31.6% (77/244) and in the control group was 25.8% (62/240). The fix-effect model was used for statistical analysis because the heterogeneity test (*I*^2^ = 0%, *p* = 0.47) suggested that there was homogeneity among the studies. A meta-analysis showed that there was no significant difference in the incidence of PDA between the colostrum group and the control group (*p* = 0.17, RR = 1.22, 95% CI = 0.92–1.62).

#### Rate of Weight Gain (kg.d)

Totally, two studies (5, 6) reported the rate of weight gain (kg.d) in 452 preterm infants. The random-effect model was used to estimate the combined result because of the strong heterogeneity among studies (*I*^2^ = 98%, *p* < 0.00001). A meta-analysis showed that there was significant difference in the rate of weight gain (kg.d) between the colostrum group and the control group (*p* < 0.00001, MD = 2.63, 95% CI = 2.10–3.16).

## Discussion

Since the concept of OAC was put forward, many scholars at home and abroad have carried out relevant research on colostrum oral immunotherapy. Most scholars apply or drip frozen colostrum after disinfection and heating, but the number and duration of daily operation are different. In the study by Glass et al. very low BW infants received oral care with either mother's own colostrum (treatment) or sterile water (control) every 3 h from the day of life 2–7 (10). For the oral care procedure, 0.2 ml of mother's colostrum or sterile water was applied to the oral mucosa by a nurse using a cotton-tipped applicator every 3 h during care times. A study by Ferreira et al. injects 0.2 ml of mother's own colostrum or sterile water into a sterile needle-free 1 ml syringe and drips 0.1 ml of colostrum or sterile water into each side of the oral mucosa (7). This procedure is performed every 2 h within 48 h (24 times), starting from 48 to 72 h after birth. In a study in India, a total of 0.2 ml of human milk or placebo (sterile water) was administered for very preterm infants every 3 h *via* the oropharyngeal route, beginning within 24 h after birth and continued until oral feeds were initiated, using a 1 ml syringe (without needle) with its tip placed between the cheek and the gum on either side (4). Therefore, although many studies have reported the safety and feasibility of OAC, there is no unified theory on the best time and frequency of colostrum oral application or drip.

Necrotizing enterocolitis is a potentially lethal gastrointestinal infection and inflammatory disorder. Preterm infants diagnosed with NEC often have a variety of long-term complications, which have caused a huge economic burden to the family and society. Therefore, the prevention of NEC is more important than the treatment of NEC (15). Current studies mostly believe that OAC may prevent NEC through the following aspects: (1) enhance the interaction between cytokines in colostrum and oropharyngeal mucosal tissues to provide systemic protective immunostimulatory effects; (2) protective biokines exert their antibacterial, anti-inflammatory, immunomodulatory, and antioxidant effects; (3) establish mucosal barrier protection against pathogens; (4) oligosaccharides prevent pathogen colonization, proliferation, and translocation not only by promoting symbiotic bacterial colonization but also by direct interaction with epithelial cells to maintain intestinal integrity; (5) anti-inflammatory effects; and (6) antioxidant effects (16). A Spanish study took 100 newborns (<32 week's gestation and/or <1,500 g) and divided them into two groups: mother's milk group (*n* = 48), receiving 0.2 ml of oropharyngeal mother's milk every 4 h for the first 15 days of life, and a control group (*n* = 52), not receiving oropharyngeal mother's milk. At the end of the study, two newborns in each group were diagnosed with NEC (Bell stage ≥ 2). The results showed that there was no significant difference in the incidence of NEC (Bell stage ≥ 2) between the two groups (17). A meta-analysis of RCTs showed that oropharyngeal colostrum therapy was not associated with a statistically significant reduction in the incidence of NEC stage ≥2 (18). Another meta-analysis included six studies that compared early oropharyngeal colostrum vs. water, saline, placebo, or donor vs. no intervention, enrolling 335 preterm infants with gestational ages ranging from 25 to 32 weeks' gestation and BWs of 410–2,500 g, the results showed no significant differences between administration of oropharyngeal colostrum and control for the incidence of NEC (19). There is no consistent result in view of the fact that OAC can reduce the incidence rate of NEC in preterm infants, so further studies are needed to confirm.

Late-onset sepsis is one of the serious diseases threatening the lives of children in the NICU, with high incidence and mortality. There are research reports that the morbidity and mortality rates of LOS are 20–38% and 13–19% (20). The mucosal barrier of preterm infants is weak. Artificial feeding, delayed feeding, and antibiotics will cause the mucosal colonization of pathogenic bacteria, various invasive operations may cause the colonized pathogens to cross the immature epithelial barrier and enter the blood to cause infection (21). Therefore, LOS is a multifactorial disease. A pilot, single-center, 1:1 parallel RCT in China shows that the incidence of LOS was lower in the OAC group, the difference was statistically significant (5). A meta-analysis showed that oropharyngeal colostrum has a trend toward downregulating the incidence of proven sepsis, but there was no significant difference in the incidence of LOS (22). One study measured interleukin (IL) IL-6, IL-8, IL-10, IL-1ra, tumor necrosis factor-alpha (TNF-α), and interferon gamma (IFN-γ) in the mother's milk group and control group at 1, 3, 15, and 30 days after birth, the results showed that preterm infants receiving OAC showed lower proinflammatory factors (IL-6, IL-8, TNF-α, and IFN-γ) and higher levels of anti-inflammatory factors (IL-10). It is suggested that OAC has a positive impact on the development of the immune system and inflammatory response. This change is beneficial to preterm infants because inflammatory response is a common upstream pathway of most perinatal diseases (17). This further shows that OAC has a positive effect on the regulation of immune system and anti-inflammatory response in preterm infants and then reduces the occurrence of LOS.

In recent years, the survival rate of newborns, especially very preterm infants and very low BW infants, has increased significantly, and their mortality has gradually increased. NEC and LOS are the main causes of preterm infant death. In a pilot prospective randomized study on preterm (<32 weeks gestation and 1,500 g weight) infants, the researcher compared oropharyngeal administration of mother's milk practice (applying 0.2 ml of mother's colostrum or milk prior to gavage feeding until full oral feeding is reached) with regular gavage feeding. It was found that oropharyngeal administration of mother's milk practice did not affect the incidence of neonatal mortality (6). VAP is one of the common nosocomial infections in the NICU. According to statistics, the incidence of VAP in NICU is as high as 42.8% and the mortality is as high as 16.4% (23). The establishment of artificial airway destroys the natural barrier of children's mouth and nasal cavity to bacteria to a certain extent. Therefore, strict and effective oral care can prevent the colonization of bacteria in the upper respiratory tract and reduce the occurrence of VAP. Colostrum is rich in a variety of immune substances, such as SIgA, lactoferrin, lysozyme, and complement. However, due to the establishment of an artificial airway, children with mechanical ventilation cannot absorb colostrum through oropharyngeal lymphoid tissue or oral mucosa, which loses the protective effect of colostrum to a certain extent. Therefore, the use of OAC may prevent pathogen colonization in respiratory and digestive tract mucosa, so as to protect the mucosal immune barrier and reduce the occurrence of VAP. Totally, 11 RCTs with a total of 1,173 children were included in this study. The results of this study showed that although OAC did not reduce mortality, the incidence of BPD, IVH (grade ≥3), PVL, ROP, and PDA in preterm infants, but it reduced the incidence of NEC, LOS, and VAP in preterm infants, shortening time to reach full enteral feeds and duration of hospital stay, increasing the rate of weight gain (kg.d).

The main conclusions of this meta-analysis are inconsistent with those of some previous meta-analyses and observational studies. The reasons may be as follows: (1) with the increasing research on OAC in recent years, medical staff is more proficient and standardized in the operation process of OAC; (2) most of the studies included in this meta-analysis are in recent 3 years, and the sample size is large, which has a great impact on the overall outcome; (3) intervention methods are slightly different: among the 11 included studies, 10 studies used oral drip to give breast milk, this method is simple to operate, but it may also damage the oral mucosa due to improper operation or reduce the ejection of colostrum due to the sucking action of preterm infants; 1 article used oral apply to give breast milk, this method is also very simple to operate, but the actual use amount cannot be estimated because the cotton swab will absorb most of the breast milk. (4) The control group was dripped or applied with different substances, such as normal saline, sterile water, nothing, and only routine care without any substances. Similarly, this study also has some limitations: (1) there are some differences in the start time and frequency of oral colostrum, and the amount of breast milk used for oral colostrum is also different, especially the duration of oral colostrum. Therefore, the intervention may not be entirely due to colostrum, which will affect the effect of colostrum oral application; (2) NEC and LOS are not unifactorial disorders and may be associated with a variety of factors, such as gestational age, BW, treatment received, and medical status of the preterm infant. Therefore, the effect of colostrum oral application on short-term and long-term outcomes in preterm infants still needs to be confirmed by long-term, large-sample, multicenter, and well-designed randomized controlled blinded studies.

In conclusion, OAC can reduce the incidence of NEC, LOS, and VAP in preterm infants, shortening the time to reach full enteral feeds and duration of hospital stay, and increasing the rate of weight gain (kg.d). Although OAC is a simple and economical nursing operation, there are still many problems in the specific operation, such as the way of using OAC, the frequency, and the amount of colostrum used. Therefore, the efficacy and safety of OAC still need to be further confirmed by more studies.

## Data Availability Statement

The original contributions presented in the study are included in the article/supplementary material, further inquiries can be directed to the corresponding author.

## Author Contributions

MH, ChunlL, HM, YuhZ, ChunzL, and DS contributed to the research, design, and analysis. MH, ChunlL, HM, YuhZ, ChunzL, DS, YayZ, YanZ, and CX contributed to the interpretation of the data and the drafting of the manuscript. All authors contributed to the article and approved the submitted version.

## Funding

This research was funded by the Natural Science Foundation of Inner Mongolia Autonomous Region (2020MS08034).

## Conflict of Interest

The authors declare that the research was conducted in the absence of any commercial or financial relationships that could be construed as a potential conflict of interest.

## Publisher's Note

All claims expressed in this article are solely those of the authors and do not necessarily represent those of their affiliated organizations, or those of the publisher, the editors and the reviewers. Any product that may be evaluated in this article, or claim that may be made by its manufacturer, is not guaranteed or endorsed by the publisher.
